# Design, Synthesis and *in Vitro* Degradation of a Novel Co-Drug for the Treatment of Psoriasis

**DOI:** 10.3390/pharmaceutics5020232

**Published:** 2013-04-17

**Authors:** Wing Man Lau, Charles M. Heard, Alex W. White

**Affiliations:** 1 School of Pharmacy, University of Reading, Whiteknights, P.O. Box 226, Reading, RG6 6AP, UK; 2 School of Pharmacy and Pharmaceutical Sciences, Cardiff University, Cardiff, CF10 3NB, UK; E-Mails: heard@cf.ac.uk (C.M.H.); whiteaw@cf.ac.uk (A.W.W.)

**Keywords:** psoriasis, dithranol, naproxen, co-drug, pro-drug, esterase

## Abstract

Psoriasis is a common, chronic and relapsing inflammatory skin disease. It affects approximately 2% of the western population and has no cure. Combination therapy for psoriasis often proves more efficacious and better tolerated than monotherapy with a single drug. Combination therapy could be administered in the form of a co-drug, where two or more therapeutic compounds active against the same condition are linked by a cleavable covalent bond. Similar to the pro-drug approach, the liberation of parent moieties post-administration, by enzymatic and/or chemical mechanisms, is a pre-requisite for effective treatment. In this study, a series of co-drugs incorporating dithranol in combination with one of several non-steroidal anti-inflammatory drugs, both useful for the treatment of psoriasis, were designed, synthesized and evaluated. An ester co-drug comprising dithranol and naproxen in a 1:1 stoichiometric ratio was determined to possess the optimal physicochemical properties for topical delivery. The co-drug was fully hydrolyzed *in vitro* by porcine liver esterase within four hours. When incubated with homogenized porcine skin, 9.5% of the parent compounds were liberated after 24 h, suggesting *in situ* esterase-mediated cleavage of the co-drug would occur within the skin. The kinetics of the reaction revealed first order kinetics, *V*_max_ = 10.3 µM·min^−1^ and *K*_m_ = 65.1 µM. The co-drug contains a modified dithranol chromophore that was just 37% of the absorbance of dithranol at 375 nm and suggests reduced skin/clothes staining. Overall, these findings suggest that the dithranol-naproxen co-drug offers an attractive, novel approach for the treatment of psoriasis.

## 1. Introduction

Psoriasis is a common skin disease for which there is no known cure. This chronic and relapsing inflammatory disease affects approximately 2% of the world population [1]. Affected individuals experience localized inflammation and scaling of the skin, often accompanied by intense itching and pain. At the cellular level skin keratinocytes undergo abnormal differentiation and hyper-proliferation. Additionally, up to 40% of psoriasis cases are associated with psoriatic arthritis, an inflammatory condition of the joints accompanied by pain and swelling. Although not life-threatening, psoriasis can have significant impacts on the sufferers’ quality of life. The etiology of psoriasis is not fully understood, but it has been established that its development can be influenced by both genetic and environmental factors, and that both immunological mechanisms and abnormal epidermal proliferation are involved [2,3,4].

Current advances in the treatment of psoriasis have focused almost exclusively on biological agents targeting the immunological pathways associated with the disease [5]. However, small molecule topical therapies, for example dithranol (also known as anthralin), topical corticosteroids and vitamin D analogs such as calcipotriol, remain important first-line treatments for psoriasis. These treatments reverse keratinocyte hyper-proliferation and regulate the inflammatory response in psoriatic skin. Despite the numerous treatments available for psoriasis, significant adverse effects, inadequate efficacy and therapeutic resistance have created the demand for better tolerated and more effective therapies. Furthermore, the high cost and production difficulties associated with biological agents suggest a clear need for novel small molecule drugs for psoriasis.

**Figure 1:**
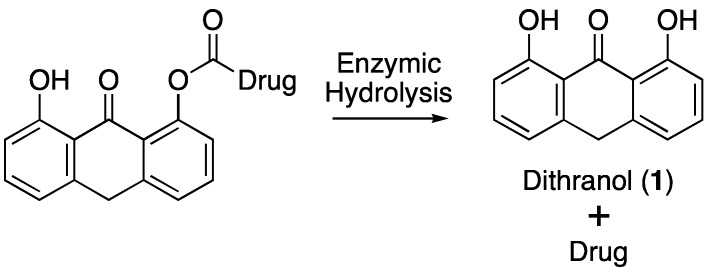
Illustration of the dithranol co-drug concept.

In the clinical management of psoriasis, topical formulations are the preferred route of drug administration. In addition, combination therapy often proves more efficacious and better tolerated than monotherapy with a single drug, although this has often been achieved with systemic agents [6]. Combination therapy could be administered in the form of a co-drug: two or more therapeutic compounds active against the same condition and linked by a cleavable covalent bond (Figure 1). The advantages of topical co-drug delivery over co-administration or co-formulation include improved drug targeting and enhanced drug stability, which have been reviewed in detail elsewhere [7].

**Figure 2:**
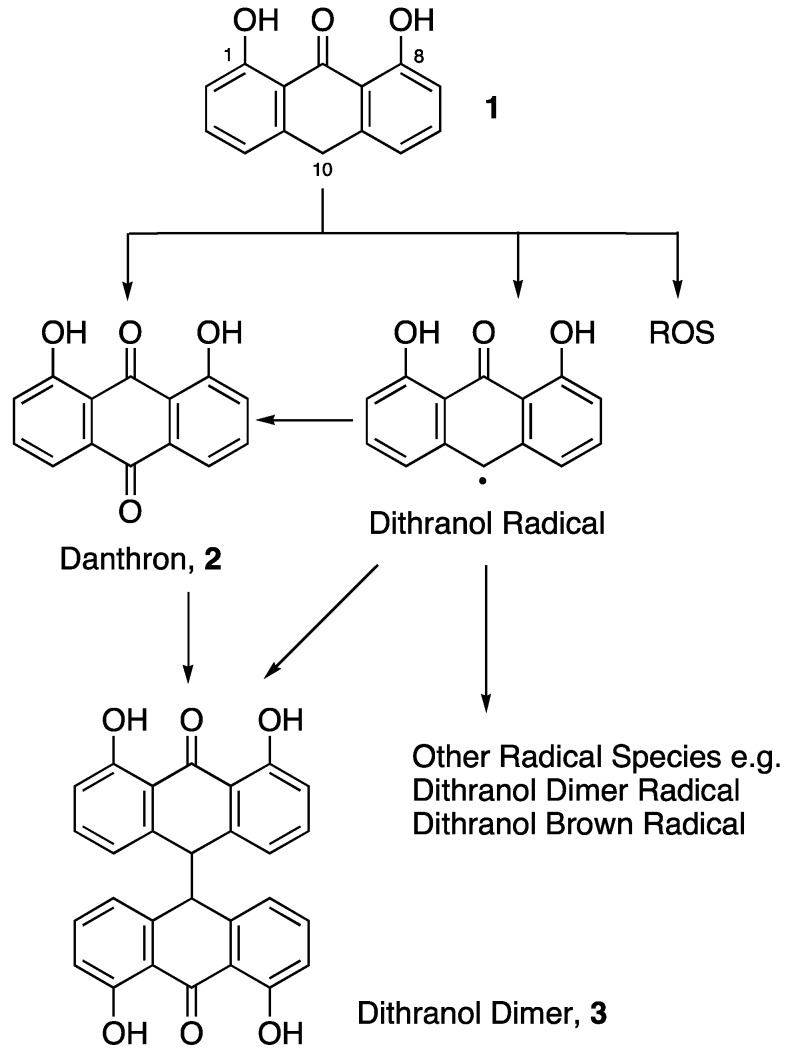
Dithranol (**1**) and its common degradation products.

Dithranol, 1,8-dihydroxyanthracen-9(10*H*)-one, (**1**), is a common and highly effective topical agent for the treatment of psoriasis. This first-line therapy is free of systemic side-effects and skin atrophy that is commonly associated with other topical treatments such as steroids. Its precise mode of action is unknown but various cellular targets and pathways have been proposed, including: DNA replication and repair mechanisms [8], the mitochondrial membrane and mitochondrial function (by inhibition of cellular respiration) [9], induction of epidermal growth factor receptor (EGFR) phosphorylation in keratinocytes [10] and modulation of several key cytosolic enzymes associated with cell proliferation and inflammation [11]. Despite its efficacy, dithranol is limited by undesirable pro-inflammatory effects on the skin, as well as severe staining of skin and clothing. Topical application of dithranol has been shown to increase the production of reactive oxygen species (ROS) in the skin [12,13]. In addition, dithranol is chemically unstable, especially when exposed to sunlight or air (or in the presence of a strong base). The skin staining effect of dithranol has been ascribed to a number of its degradation products, most notably danthron (**2**) and a dithranol dimer (**3**) as outlined in Figure 2. These properties of dithranol limit patient acceptability and ultimately its usefulness as a topical agent.

In co-drug design, the selection of therapeutic moieties is restricted to those with complementary functional groups which can form a biologically labile bond [7]. Several dithranol derivatives have been prepared and studied, mostly involving modification at the C-10 methylene group with the aim of diminishing oxygen-radical formation, reducing staining or irritation, and/or improving anti-proliferative properties [12,14,15,16]. Given the clinical efficacy of dithranol and potential for derivatization with biologically labile ester functional groups at the C-1 and C-8 hydroxyl groups, carboxylic acid containing drugs with clinical applications in psoriasis that could be formulated as a dithranol ester co-drug were investigated. In many cases, linking two active species together increases the molecular weight of the co-drug beyond the topical delivery optimum of 500; similarly the logP of the co-drug may deviate from the ideal range of approximately 1–3.5. These parameters were taken into account when considering appropriate candidates for a topical co-drug [7,17,18,19]. To evaluate the potential of a novel small molecule approach to psoriasis, a series of co-drugs based on dithranol were synthesized and the evaluation of *in vitro* bioactivation of the most promising candidate molecule was also studied herein.

**Figure 3:**
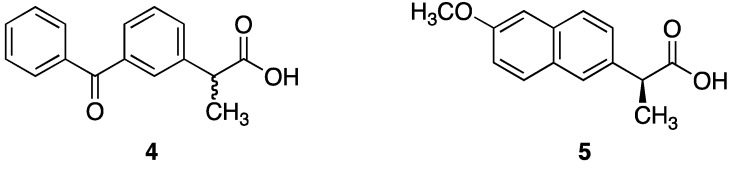
Chemical structures of ketoprofen (**4**), (*S*)-naproxen (**5**).

Non-steroidal anti-inflammatory drugs (NSAIDs) are often part of the first-line treatment for psoriasis and psoriatic arthritis [6]. NSAIDs owe their anti-inflammatory actions to the inhibition of cyclooxygenase enzymes, which are up-regulated in inflammatory disorders. Topical NSAID therapy can deliver therapeutic concentrations of the drug to the site of action and is potentially safer and more effective than oral delivery, particularly by reducing gastrointestinal side effects [20,21]. Ketoprofen (**4**) and (*S*)-naproxen (**5**) (Figure 3), both potent NSAIDs widely prescribed for inflammatory skin conditions, were identified as dithranol co-drug candidates with complementary functional groups for co-drug synthesis [22,23].

## 2. Materials and Methods

Dithranol was purchased from BUFA Pharmaceutical Products (Uitgeest, Holland). Naproxen, ketoprofen, porcine liver esterase, Hanks’ balance salt, 1,8-dihydroxyanthraquinone (danthron), iron III chloride and acetic acid were purchased from Sigma-Aldrich Company Ltd. (Poole, UK). All other chemicals and HPLC-grade solvents were obtained from Fisher Scientific (Loughborough, UK) and used without further purification. Petrol refers to petroleum ether 60–80. Kieselgel 60 F254 plates were obtained from Merck, Darmstadt, Germany. Porcine ears were obtained from a local abattoir prior to steam cleaning and were used within 3 h of slaughter.

### 2.1. General Chemical Procedures

TLC was performed on commercially available Merck Kieselgel 60 F254 plates and visualized using UV (254 nm or 366 nm). Column chromatography was performed using glass columns filled with silica gel 60 slurry under medium pressure using a hand pump. ^1^H NMR and ^13^C NMR spectra were recorded on a Bruker Avance DPX500 spectrometer with operating frequencies of 500 and 125 MHz. All ^13^C NMR spectra were proton decoupled and all spectra were obtained in deuterated chloroform (CDCl_3_). Melting points were performed in triplicate using Gallenkamp melting point apparatus (Loughborough, UK) and were not corrected. High and low resolution MS (HRMS and LRMS) were performed by the EPSRC National Mass Spectrometry Service, Swansea University, UK, using the stated ionisation method. Elemental analyses, performed by Medac Ltd. (Surrey, UK) were used to confirm compound purity (≥95%). Calculated logP (ClogP) values were determined using ChemDraw Ultra 10.0, CambridgeSoft, Cambridge, United States.

#### 2.1.1. Method 1: Acid Chloride Synthesis

Carboxylic acid derivatives of **4** or **5** were dissolved in dry tetrahydrofuran (*w*/*v* 75 mg/10 mL) and cooled to 0 °C under nitrogen. Thionyl chloride (5 equiv.) was added slowly with stirring followed by three drops of dimethyl formamide. The mixture was allowed to warm to ambient temperature and was stirred overnight. Solvents were removed under vacuum and the acid chloride product was used immediately with no further purification.

#### 2.1.2. Method 2: Dithranol Di-Ester Co-Drug Synthesis

**1** (500 mg, 1 equiv.) was dissolved in dry tetrahydrofuran (30 mL) and cooled with dry ice/acetone for five minutes with constant agitation. Pyridine (0.27 mL, 1.5 equiv.) was added dropwise under nitrogen. The appropriate acid chloride (2 equiv.) was dissolved in dry tetrahydrofuran (2 mL), cooled (dry ice/acetone) for 5 min, then added slowly into the mixture. The reaction was allowed to return to room temperature slowly and stirred overnight at room temperature. One mole per liter HCl (50 mL) was added and the volatiles were removed by rotary evaporation. The mixture was extracted with dichloromethane (2 × 30 mL). The combined organic phases were washed with saturated NaHCO_3_ solution (30 mL), dried over MgSO_4_, and purified using flash column chromatography using dichloromethane 100% through to dichloromethane:ethyl acetate:petrol 13:1:6 as eluent.

#### 2.1.3. Method 3: Dithranol Mono-Ester Co-Drug Synthesis

**1** (400 mg, 1 equiv.) was dissolved in 10 mL anhydrous hexamethylphoramide (HMPA) and chilled to 0 °C under nitrogen. The appropriate acid chloride (1 equiv.) was dissolved in dry HMPA (3 mL), cooled to 0 °C, and was added in dropwise fashion to the dithranol solution. The mixture was slowly warmed to ambient temperature and allowed to stir for 5 h. The mixture was poured into 300 mL water and extracted with dichloromethane (3 × 40 mL). The combined organic phases were washed with water (3 × 100 mL) followed by saturated NaHCO_3_ solution (100 mL). The organic phase was further washed with water (2 × 100 mL) and dried over MgSO_4_, before purification using flash column chromatography.

#### 2.1.4. Dithranol Dimer Synthesis (**3**)

Prepared according to a published procedure [24], **1** (1 g) was dissolved in boiling acetic acid (100 mL), degassed and shielded from light. 10% FeCl_3_ in acetic acid (12 mL) was added slowly. Water (5 mL) was added and the reaction product crystallized over a period of a few hours at room temperature. Re-crystallisation using acetic acid afforded the desired product as a green powder (602 mg, 30%). mp 246–247 °C; ^1^H NMR (CDCl_3_) δ 4.61 (s, 2H), 6.40 (d, 4H, *J* = 7.4 Hz), 6.93 (d, 4H, *J* = 8.3 Hz), 7.41 (t, 4H, *J* = 8.0, 7.8 Hz), 11.73 (s, 4H, OH). ^13^C NMR (CDCl_3_) δ 56.3, 116.7, 117.1, 119.5, 135.7, 141.1, 162.0, 192.2.

### 2.2. Co-Drug Synthesis

#### 2.2.1. Synthesis of Dithranol Di-Naproxen Co-Drug (**6**): 9-*Oxo*-9,10-dihydroanthracene-1,8-diyl bis(2-(6-methoxy-naphthalen-2-yl)propionate)

Prepared by *Method 2*, the co-drug was recovered as pale yellow crystals (292 mg, 30%). mp 90–95 °C; ^1^H NMR (CDCl_3_) δ 1.76 (d, 6H, *J* = 7.1 Hz), 3.85 (s, 6H), 4.24 (s, 2H), 6.77 (d, 2H, *J* = 8.0 Hz), 7.07 (s, 2H), 7.09 (m, 2H), 7.18–7.37 (m, 6H), 7.51 (d, 2H, *J* = 8.4 Hz), 7.68 (m, 4H), 7.74 (s, 2H); ^13^C NMR (CDCl_3_) δ 17.9, 32.4, 44.8, 54.5, 104.8, 118.1, 121.4, 124.8, 125.1, 125.5, 125.8, 126.4, 128.2, 128.6, 132.1, 133.0, 134.7, 140.5, 149.6, 156.9, 172.5, 181.1; HRMS (ES+) *m*/*z* 673.2204 [M + Na]^+^ calcd. 673.2197 for C_42_H_34_O_7_Na.

#### 2.2.2. Synthesis of Dithranol Di-Ketoprofen Co-Drug (**7**): 9-*Oxo*-9,10-dihydroanthracene-1,8-diyl bis(2-(3-benzoylphenyl)propionate)

Prepared by *Method 2*, the co-drug was obtained as a pale yellow solid (479 mg, 39%). mp 70–75 °C; ^1^H NMR (CDCl_3_) δ 1.80 (d, 6H, *J* = 7.0 Hz), 4.23 (q, 2H, *J* = 7.3 Hz), 4.36 (s, 2H), 6.92 (d, 2H, *J* = 6.9 Hz), 7.34 (d, 2H, *J* = 7.6 Hz), 7.47–7.76 (m, 14H), 7.85 (d, 4H, *J* = 7.7 Hz), 7.93 (s, 2H); ^13^C NMR (CDCl_3_) δ 18.8, 33.17, 45.5, 122.1, 125.4, 126.2, 128.4, 128.7, 129.1, 129.2, 129.5, 130.1, 132.0, 132.5, 132.6, 133.1, 137.6, 138.1, 140.7, 141.5, 150.3, 172.5, 181.8, 196.5; MS (CI+) *m*/*z* 699.4 [M + H]^+^, 716.4 [M + NH_4_]^+^; MS (FAB) *m*/*z* 699.3 [M + H]^+^, 698.3 [M]^+^.

#### 2.2.3. Synthesis of Dithranol Mono-Naproxen Co-Drug (8): 8-Hydroxy-9-*oxo*-9,10-dihydroanthracen-1-yl 2-(6-methoxynaphthalen-2-yl)propanoate

Prepared by *Method 3*, using ethyl acetate/hexane gradient (from 10% to 20% ethyl acetate) for column chromatography, the co-drug was obtained as a pale-yellow solid (357 mg, 46%), mp 146–148 °C; ^1^H NMR (CDCl_3_) δ 1.73 (d, 3H, *J* = 7.2 Hz), 3.84 (s, 3H), 4.22–4.26 (m, 3H), 6.73–7.18 (m, 6H), 7.33 (t, 1H, *J* = 7.9 Hz), 7.40 (t, 1H, *J* = 7.9, 7.8 Hz), 7.51 (dd, 1H, *J* = 1.7, 8.4 Hz), 7.66–7.76 (m, 2H), 7.76 (s, 1H), 12.68 (s, 1H); ^13^C NMR (CDCl_3_) δ 18.6, 32.9, 45.7, 55.3, 105.7, 115.4, 117.1, 118.1, 119.0, 122.3, 123.6, 126.4, 126.7, 126.5, 127.2, 129.1, 129.4, 133.8, 134.0, 135.4, 135.5, 140.6, 143.0, 151.3, 157.7, 163.1, 173.5, 188.9; HRMS (ES+) *m*/*z* 439.1541 [M + H]^+^ calcd. 439.1540 for C_28_H_23_O_5_.

#### 2.2.4. Synthesis of Dithranol Mono-Ketoprofen Co-Drug (**9**): 8-Hydroxy-9-*oxo*-9,10-dihydroanthracen-1-yl 2-(3-benzoylphenyl)propanoate

Prepared by *Method 3*, using ethyl acetate/petrol with 5% CH_2_Cl_2_ (gradient from 10% to 20% EtOAc) for column chromatography, the co-drug was an orange solid (302 mg, 37%). mp 60–61 °C; ^1^H NMR (CDCl_3_) δ 1.68 (d, 3H, *J* = 7.1 Hz), 4.14–4.17 (m, 3H), 6.69–6.73 (m, 2H), 6.80 (d, 1H, *J* = 7.8 Hz), 7.16 (d, 1H, *J* = 7.7 Hz), 7.28 (t, 1H, *J* = 7.8 Hz), 7.34–7.74 (m, 9H), 7.86 (s, 1H), 12.5 (s, 1H); ^13^C NMR (CDCl_3_) δ 18.5, 32.9, 45.6, 115.4, 117.0, 118.1, 122.3, 123.4, 125.6, 126.6, 128.3, 128.3, 128.6, 129.2, 129.4, 129.7, 130.1, 131.9, 132.1, 132.5, 134.1, 135.6, 137.6, 138.0, 140.6, 143.1, 151.1, 172.9, 188.8, 196.5.

### 2.3. HPLC Analysis

A HPLC method was developed to simultaneously analyze the mono-naproxen co-drug **8**, along with the parent compounds dithranol (**1**) and naproxen (**5**), and the dithranol derivatives **2** and **3**. Samples were analyzed at ambient temperature using an Agilent 1100 series automated system with a quaternary solvent delivery system and a variable wavelength detector. The instrument was fitted with a Gemini C_18_, 5 μm, 250 × 4.6 mm column (Phenomenex, Macclesfield, UK) and a Phenomenex Securityguard pre-column. The wavelength was set at 230 nm with a flow rate of 1 mL·min^−1^ and the injection volume was 100 μL. Two mobile phase compositions were used; mobile phase A was deionised water (adjusted with H_3_PO_4_ to pH 2.2) and mobile phase B was MeCN. A gradient mobile phase starting with H_2_O/H_3_PO_4_ (60:40) for 6.5 min, changing to H_2_O/H_3_PO_4_ (10:90) over 1 min, with the same condition running for 12.5 min and then returning to the initial conditions over 3.5 min. Calibration curves for each compound was constructed using the mobile phase and each provided *R*^2^ of >0.999. The retention times for **5**, **2**, **1**, **3** and **8** were 6.5, 11.7, 12.4, 16.7 and 17.3 min respectively. The limits of detection (LoD) were 0.008, 0.45, 0.09, 1.8 and 0.9 μg·mL^−1^ respectively.

### 2.4. Spectrophotometric Analysis

Dithranol **1** and the co-drug **8** were diluted in 5 mL of MeCN to produce an equimolar (50 μM) solution of each and measured quantitatively using a Hewlett Packard 8452A diode array spectrophotometer with 1 cm quartz cells, scanning from 190 to 1000 nm. The absorbance at 375 nm was recorded and all UV spectrophotometry experiments were carried out in triplicate.

### 2.5. Enzymatic Co-Drug Hydrolysis

#### 2.5.1. Hydrolysis Using Porcine Liver Esterase

Co-drug **8** was dissolved in acetonitrile at five concentrations, 91, 80, 69, 34 and 29 μM and incubated with 120 IU mL^−1^ of porcine liver esterase (PLE) in phosphate buffered saline (PBS) and 5% acetonitrile to give a total reaction volume of 10 mL. A magnetic stirrer was added and the reaction medium was constantly stirred. The solution was maintained at 25 °C. At regular intervals 400 μL was withdrawn and 400 μL of quenching solution (80% acetonitrile and 20% deionised H_2_O adjusted to pH 2.2 with H_3_PO_4_) was added. Samples were centrifuged at 12,000 rpm for 15 min, the supernatant was sampled and analyzed by HPLC. Control experiments contained **8** in an identical medium with the absence of PLE. The reactions were preformed in triplicate.

#### 2.5.2. Hydrolysis Using Porcine Skin Homogenate

Freshly excised porcine ears were immersed in Hanks buffer with ice during transport, before being washed with running tap water. Full thickness skin was isolated from underlying cartilage by blunt dissection using a scalpel. Hairs were removed with electric clippers. Skin samples (4 × 2 g) were cut into small pieces and placed in 15 mL PBS, before being homogenised using a high-shear laboratory mixer (Silverson Machines Ltd., UK) for 1 min. Co-drug **8** was first dissolved in an appropriate amount of acetonitrile to produce a final reaction solution with 80 μM of **8** in 2.5% acetonitrile in PBS. All five vials and two control experiments lacking porcine skin homogenate (PSH) were placed in an incubator set at 32 °C (average surface skin temperature). Samples of 400 μL were periodically taken and the reaction was terminated by adding an equal volume of quenching solution (as PLE method). The mixture was then centrifuged for 15 min at 14,000 rpm, the supernatant was collected and analyzed by HPLC.

## 3. Results and Discussion

### 3.1. Co-Drug Synthesis

The preparation of ester co-drugs was not as straightforward as expected due to the reactivity of the C-10 methylene group in **1**. Although the majority of reported dithranol analogs are modified at C-10, a limited number 1-*O*-mono-substituted and 1,8-*O*-disubstituted esters have been reported [16,25]. The published synthetic methods failed to yield the anticipated dithranol ester derivatives in our hands, instead yielding C-10 substituted derivatives (data not shown). On the basis of these observations, alternative preparative routes for dithranol esters were investigated. The di-ester co-drugs **6** and **7** were successfully prepared by conversion of each NSAID carboxylic acid to an acid chloride. Cooling the acid chloride to −78 °C for 5 min prior to reaction with **1** proved essential for di-ester formation. The preparation of the dithranol monoester co-drugs **8** and **9** required cooling each acid chloride to 0–5 °C before addition to **1** and in addition to that hexamethylphosphoramide (HMPA) proved to be the only effective solvent. Many alternative solvents were investigated, but none yielded the required co-drug product.

### 3.2. Co-Drug Selection

The liberation of parent moieties post-administration, by enzymatic and/or chemical mechanisms, is clearly a pre-requisite for an effective co-drug. PLE is commonly used as a model enzyme for cutaneous metabolism to assess the enzymatic hydrolysis of pro-drugs or co-drugs [23,26,27]. Table 1 illustrates the successfully synthesized co-drug candidates and summarises some of their physicochemical properties. Considering the diester co-drugs, **6** and **7** proved to be labile to *in vitro* PLE enzymatic hydrolysis. It was envisioned that the two ester bonds of **6** and **7** would be cleaved by exhaustive esterase activity to liberate **1** as well as **5** and **4** respectively. However, HPLC analysis revealed the formation of additional, unidentified metabolites suggesting a more complicated degradation pathway than predicted. Furthermore, the high molecular weights of the diester co-drugs, and, as a corollary, their ClogP values, were not considered ideal for topical delivery. Hence they were not selected for further investigation in this study.

Out of the two mono-ester co-drugs, **8** possessed a more suitable physicochemical properties with a relatively low molecular weight and near optimal lipophilicity (MW = 438 and ClogP = 5.45) for delivery via the skin. Hence it was chosen for further study.

**Table 1:** Summary of dithranol-based ester co-drugs.

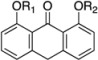					
Cpd.	MW ^a^	R_1_	R_2_	Synthetic Yield (%)	ClogP ^b^
**6**	650	I 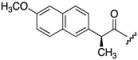	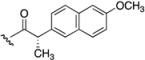	30	8.54
**7**	698	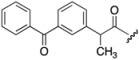	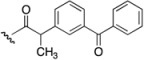	39	9.74
**8**	438	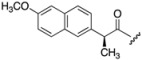	H	46	5.45
**9**	462	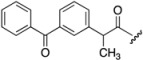	H	37	5.56

^a^ MW = Molecular weight; ^b^ ClogP = calculated logP, determined using CambridgeSoft ChemDraw Ultra; the reported value is the average of three different fragmentation methods.

### 3.3. Hydrolysis of Dithranol-Naproxen Co-Drug (8)

**Figure 4:**
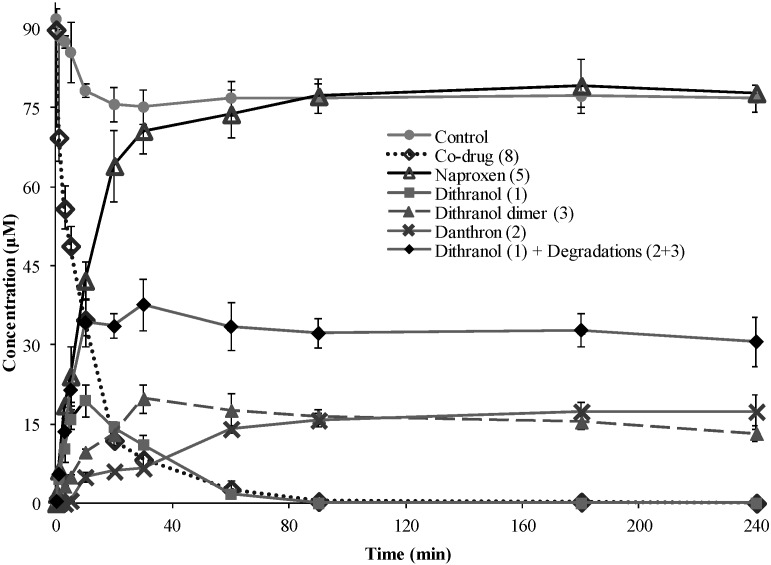
Porcine liver esterase (PLE) hydrolysis profile of co-drug **8** from an initial concentration of 91 μM (mean ± s.d., *n*=3). The graph shows disappearance of **8** and corresponding appearance of the parent compounds, **5** and **1**, over time in the presence of PLE. **2** and **3** were also detected. The total amount of **1** plus its degradation products (**2** and **3**) is shown. All data are plotted against the control experiment without PLE, which showed negligible amount of co-drug hydrolysis.

**Figure 5:**
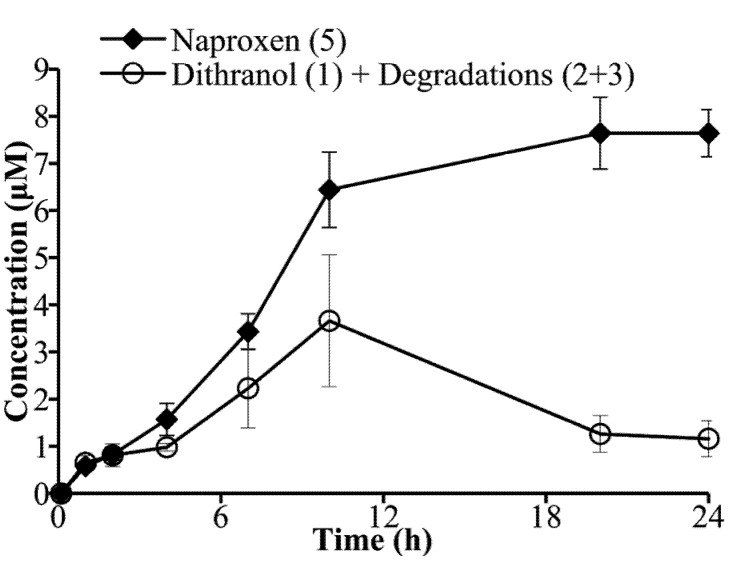
Liberation of the parent compounds, **1** and **5** plus degradation products (**2** and **3**), from co-drug **8** (starting concentration 80 μM), in the presence of fresh porcine ear skin homogenate (mean ± s.d., *n*= 4).

Hydrolysis of **8** was investigated by incubation with PLE to confirm that the co-drug was a substrate for esterase hydrolysis (Figure 4), and by treatment with PSH to evaluate hydrolysis in whole skin tissue (Figure 5). Porcine tissue is established as a reliable model for human skin [28,29].

The PLE experiment was performed at 25 °C to reduce the rate of enzymatic hydrolysis to a velocity which could be easily measured compared to physiological temperature. In the control experiments, with co-drug **8** in reaction medium without PLE, the parent compounds (**5** and **1**) were below the limit of detection (LoD), indicating that no chemical hydrolysis had occurred. Following incubation with PLE, the co-drug was fully hydrolyzed within 4 h, suggesting that **8** is a substrate for PLE which is responsible for the hydrolysis of the co-drug (Figure 4). Since the co-drug comprises a 1:1 molar ratio of **1** and **5**, equimolar amounts of the parent compounds should be liberated and detected upon cleavage of the ester bond. The rate of co-drug disappearance correlates well with the rate of appearance of **5**, and the release was rapid and complete. In contrast, the proportional increase was not seen for **1** (or its degradation products) after the initial stage. This is probably due to further oxidation of danthron (**2**) and dithranol dimer (**3**) to compounds that could not be identified in this experimental setting, for example dithranol brown and various anthraquinone derivatives [13].

The hydrolysis of **8** was also investigated using freshly excised and homogenized whole pig skin. This model provides physiologically relevant conditions to study the degradation of **8** in the presence of total skin enzymes, providing an indication of co-drug efficacy within human skin *in vivo*. In a control experiment, **8** was relatively stable in the reaction medium alone (2.5% acetonitrile in PBS) at room temperature. Under these control conditions, the co-drug degradation products were below LoD after 24 h, indicating that the co-drug did not undergo non-enzymatic hydrolysis (data not shown). Following 24 h PSH treatment, 7.6 ± 0.5 μM of **5** (9.5% of the initial co-drug concentration) and 1.16 ± 0.38 μM of **1**, alongside its degradation products, were detected from a starting concentration of 80 μM of **8** (Figure 5). Comparing these results against the control, PSH-induced hydrolysis within the same timeframe can be attributed to hydrolysis by skin enzymes. The quantification of **5**, a stable drug liberated from **8** was the most reliable indicator of co-drug hydrolysis. It has been explained above that the liberation rate of **5** did not match that of **1** (plus the detectable degradation products) probably attributed to dithranol degradation also yielded products that were not detected by the current analytical HPLC method. This discrepancy does not detract from the results, since such degradation is normal and expected of dithranol. The production of **5** was lower in the whole skin (PSH) experiment compared to the enzyme (PLE) experiment. This may reflect a lower enzyme concentration or reduced substrate specificity for the porcine skin enzymes.

### 3.4. Co-Drug Hydrolysis Kinetics

**Figure 6:**
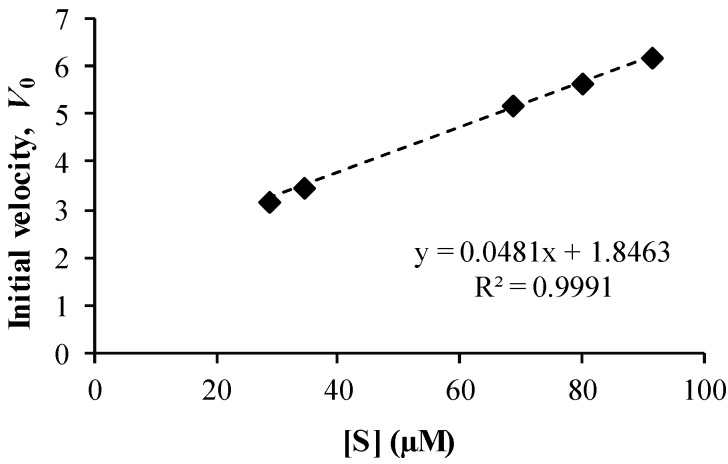
Dependence of initial velocity (*V*_0_, μg·min^−1^) on co-drug (**8**) substrate concentration [S] in a PLE hydrolysis experiment (*n* = 3).

**Figure 7:**
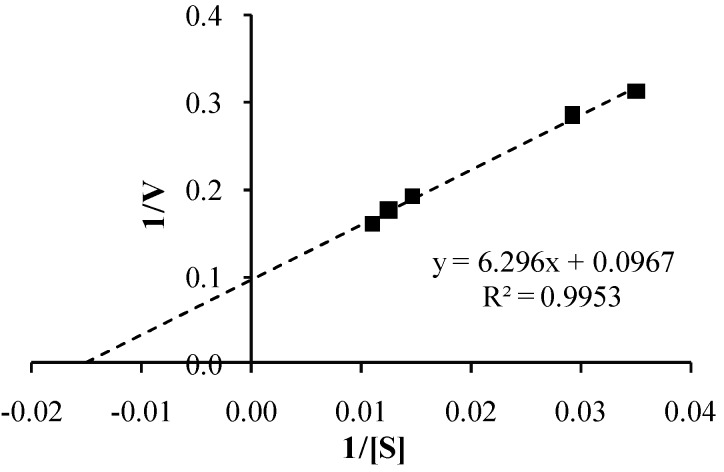
Lineweaver-Burk plot derived from a PLE hydrolysis experiment with co-drug **8** as substrate, S (*n* = 3).

The kinetics of the PLE-catalysed hydrolysis of co-drug **8** by PLE was analyzed using the Michaelis-Menten model. The initial velocity, *V*_0_, was calculated by determining the gradient of naproxen liberation in the first 5 min of the reaction, at a range of concentrations of **8**. The linear relationship obtained by plotting *V*_0_ *versus* substrate concentration [S] (Figure 6) suggests that the reaction was first order with rate constant, *k* = 0.048 min^−1^. A Lineweaver-Burk plot was constructed (Figure 7), from which the maximal velocity (*V_max_* = 10.3 ± 0.14 μM·min^−1^) and the Michaelis constant (*K_m_* = 65.1 ± 0.99 μM) were obtained (both parameters expressed as mean ± s.d.).

It was also noted that the PLE hydrolysis kinetic data for co-drug **8** are similar to those previously reported for other topical co-drugs, including retinyl ascorbate (*V_max_* = 0.28 μM·min^−1^; *K_m_* = 1430 μM) and retinyl-2-carboxy-2-hydroxyethanoate (*V_max_* = 1.3 μM·min^−1^; *K_m_* = 860 μM) [26]. In comparison with these data, the lower *K_m_* and higher *V_max_* values for co-drug **8** suggest that the co-drug is an effective substrate for PLE.

### 3.5. Spectrophotometric Analysis

**Figure 8:**
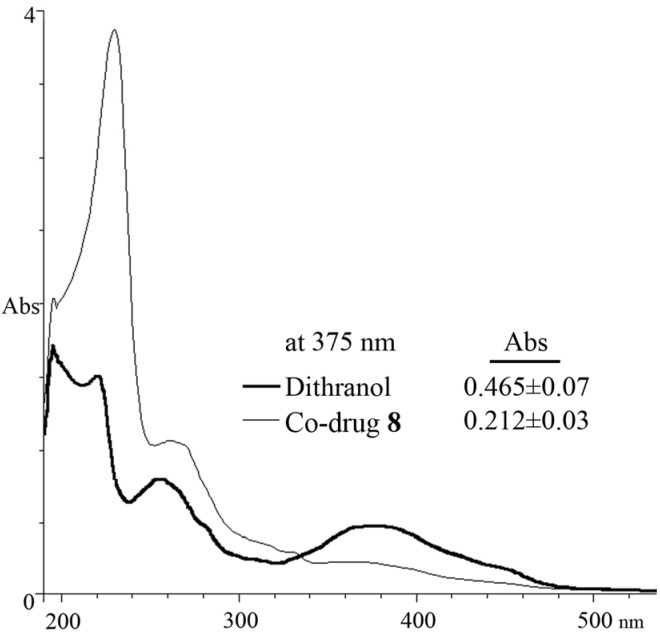
UV spectrum comparison of 50 μM dithranol (**1**) and 50 μM co-drug **8** in MeCN (Abs at 375 nm is mean ± s.d., *n* = 3).

A benefit of the dithranol co-drug approach is the administration of a modified chromophore with improved absorption and colouration properties. This is a significant consideration in the clinical use of dithranol, since intense staining of skin and clothing are unpleasant side-effects of this otherwise highly efficacious drug [30]. Furthermore, the structural modification of dithranol may confer chemical stability by reducing auto-oxidation. The modulation of colour intensity was confirmed spectrophotometrically (Figure 8). Our data showed that under equimolar concentrations, dithranol is 63% more colour intense than **8**. From this experiment, it can be hypothesised that the severe discolouration of skin and clothing due to dithranol could be significantly reduced. This hypothesis was in-line with the reduction in skin staining seen when applied to porcine skin compared to **1** [17].

## 4. Conclusion

A novel ester co-drug of dithranol and naproxen (**8**), comprising anti-proliferative and anti-inflammatory moieties for the treatment of psoriasis, has been synthesized and evaluated. The lack of reproducibility of published methods for preparing dithranol esters prompted the development of a new synthetic route to these compounds, reported herein. Co-drug **8** was selected for further investigation where hydrolysis experiments demonstrated that **8** is an effective substrate for porcine liver esterase and that enzymic hydrolysis of the co-drug was complete in 4 h. Although co-drug hydrolysis was less efficient in the presence of homogenized porcine skin, the results are promising, since hydrolysis would be expected to be substantially increased *in situ* within non-excised, viable skin. Spectrophotometrically, the colour of the co-drug is less intense than the parent compound dithranol at visible wavelengths. The results from these studies support the potential value of the co-drug approach as a novel treatment modality for psoriasis and that the dithranol-naproxen co-drug warrants further investigation as a novel treatment for psoriasis.
